# Investigation of XLPE Cable Insulation Using Electrical, Thermal and Mechanical Properties, and Aging Level Adopting Machine Learning Techniques

**DOI:** 10.3390/polym14081614

**Published:** 2022-04-15

**Authors:** Priya Selvamany, Gowri Sree Varadarajan, Naresh Chillu, Ramanujam Sarathi

**Affiliations:** 1 Division of High Voltage Engineering, Department of Electrical and Electronics Engineering, College of Engineering, Guindy Campus, Anna University, Chennai 600025, India; priya.gdv@gmail.com; 2 Department of Electrical Engineering, Indian Institute of Technology Madras, Chennai 600036, India; ee17d304@smail.iitm.ac.in (N.C.); rsarathi@iitm.ac.in (R.S.)

**Keywords:** aging, diffusion coefficient, trap depth, LIBS, XLPE, machine learning, PCA, neural networks

## Abstract

Hydrothermal and chemical aging tests on a 230 kV cross-linked polyethylene (XLPE) insulation cable were carried out in the present study to evaluate the degradation and aging levels qualitatively. The samples were subjected to water aging at a temperature of 80 °C, and in an aqueous ionic solution of CuSO_4_ at room temperature. The diffusion coefficient results indicated that the ion migration was not at the same rate in the aging conditions. The diffusion coefficient–D–of the sample immersed in an aqueous CuSO_4_ solution was lower than the hydrothermally aged specimens. The hydrophobicity of aged specimens decreased considerably compared to unaged samples. The distribution of trapped charges was quantitatively characterized. The presence of shallow trap energy states were observed in unaged XLPE, whereas the deep trap sites were noticed in aged specimens. In addition, the charge trap characteristics were different for positive and negative charge deposition. Various material characterization techniques, viz. dynamic mechanical analysis (DMA), tensile, contact angle, and LIBS, were further employed on the aged and virgin specimens. The tensile behavior of the hydrothermally aged specimen was degraded due to the oxidised regions, which had formed a weak spot against the mechanical stress. Reduced glass transition temperature and increased loss tangent measurements were noticed for aged specimens over their unaged counterparts. Machine learning techniques, such as the principal component analysis (PCA) and the artificial neural network (ANN) analysis, were performed on LIBS spectral data of the samples to classify the aging mechanisms qualitatively.

## 1. Introduction

Cross-linked polyethylene (XLPE) cables are critical components of contemporary electrical power networks. Their dependability and endurance are heavily influenced by the level of aging of the cable insulation. Under normal working conditions, the deterioration of insulating materials is the most serious issue for underground power cables [1,2]. The cable is prone to thermal aging, which can cause permanent damage to the insulation during operation. Phillips indicated that the thermal aging of XLPE at operating temperatures causes a considerable increase in the nucleation density and growth rates of bow-tie trees [3]. Thermal aging can cause XLPE insulation’s crystal structure to alter, resulting in severe loss of its thermal, mechanical, and dielectric characteristics [4,5]. To address these concerns, several researchers have focused on physicochemical studies to analyze the morphological changes in XLPE insulation due to aging.

Garton et al. discovered that ionic impurities in cable insulation increase water tree initiation [6]. These pollutants in the insulation material may migrate from the shield material or be introduced from the outside environment [7]. Qureshi discussed the existence of salts in soils such as sulfide, chlorides, and nitrates [8]. Since the cables are buried directly in soils that may contain a wide range of chemical species, diffused ions can contaminate the cable insulation.

Furthermore, the copper sulfate solution was discovered in the location of the electrochemical tree. Copper sulfate solution is formed when a copper conductor corrodes in the presence of water [9]. The greater acidity strength caused by CuSO_4_ at 50 °C was shown to be more effective in spreading water treeing over the XLPE insulation [10]. Electrical, thermal, mechanical, and environmental stresses all substantially influence the life of an insulation system. Thus, cable life predictions are frequently unexpected, owing to multi-stress aging.

Mechanical stresses in polymeric cable insulation can be caused by residual internal tension present during manufacturing and locked in as the cable cools, or thermo-mechanical stresses caused by differential thermal expansion between conductor and insulation [10]. Thus, it is worthwhile to investigate the viscoelastic characteristics of cable polyethylene in order to have a better understanding of its microstructure. As a result, it is necessary to investigate how mechanical stress and strain affect the electrical performance of XLPE insulation. The link between the microstructure and physicochemical parameters of XLPE insulation during hydrothermal and CuSO_4_ aging must be investigated further, especially for higher voltage insulation cables. The contact angle, which determines surface wetting, is the most prominent technique because it provides information on microscopic chemical inhomogeneity along the polymer’s surface after aging [11].

Moreover, the charge accumulated on the insulating material surface due to steady contact with the current-carrying conductor may alter the surface trap distribution. Therefore, the resultant stresses lead to the degradation of the insulating material. The surface potential and charge trap characteristics of the insulating material give a quantitative description to ensure its reliability and service life [12]. The DMA method accurately examines the relaxation of molecular chains under temperature variation. DMA tests as well as tensile studies are to be studied prior to the usage of the materials in real-time conditions.

Water or OH groups in a polymer, as well as metal oxidation, play an important role in insulation deterioration. LIBS (Laser Induced Breakdown Spectroscopy) is an advanced technique for determining the elemental analysis and has the advantage of remote analysis as well as multi-elemental detection [13]. Many soft computing approaches have been used for the LIBS spectral data for plastic identification [14,15]. Boueri et al. recently assessed the effective plastic identification rates ranging from 81 to 100% by employing artificial neural networks (ANNs) with LIBS [15]. The majority of the work documented in the literature utilizing LIBS has mostly focused on identifying various types of polymers and ionic species present in the damaged cable. To the best of our knowledge, there are very few studies concerning the application of LIBS for electrical cable degradation examination. Thus, the study proposes to investigate the level of aging qualitatively, which might lead to a better understanding of aging and degradation mechanisms, which is a significant contribution of the current work.

The present study is focused on the XLPE aging scenario and subsequently investigated their electrical and mechanical characteristics suitable for insulation. Specifically, methodical experimental studies were carried out to understand the following important aspects: (i) experimental investigation on the contact angle of virgin and aged XLPE, (ii) variation in contact angle on the specimen due to water droplet, (iii) the surface charge and trap characteristics under DC voltage, (iv) the characteristic variation in mechanical properties over the different aged through DMA studies, and (v) ranking the performance of aged XLPE specimen through the LIBS technique.

## 2. Materials and Methods

### 2.1. Materials

The samples for this investigation were cut from 230 kV, 1200 mm^2^ XLPE cable insulation from TANGEDCO Porur (GIS) site, Chennai, with a cable cutting tool. For the aging studies, copper sulfate (CuSO_4_) solution and water were used. For XLPE aging investigations, a 1 M solution of copper sulfate was added to distilled water, and the samples were immersed in the resultant solution at room temperature. Hydrothermal aging involves submerging XLPE specimens in distilled water and heating them to 80 °C. The weight of test specimens was checked on a regular basis until the weight gain remained unchanged for fluid diffusion studies.

### 2.2. Contact Angle Analysis

The contact angle of XLPE samples was measured using the goniometer through the half-angle approach. The contact angle was measured at ten different places and averaged by placing a water droplet of 20 µL on the XLPE sample surface. The half-angle approach was employed to measure the contact angle. Equation (1) specifies the contact angle measurement based on the drop’s height (H) and the base radius (R) [16].

(1) $$\theta =2\tan^{-1}\left(\frac{H}{R}\right)$$

### 2.3. Mechanical Analysis

The tensile test was conducted at room temperature with a 4 mm/min strain rate on dumbbell-shaped specimens using a universal testing machine (KIC-2-0200-C) with a loading capacity of 20 kN. The results of five samples were averaged for accuracy and reliability. The storage modulus (E’), loss modulus (E”), and damping factor (tan δ) of the investigated materials were measured using a dynamic mechanical analyzer (DMA 242 D (NETZSCH)). The DMA test was employed on rectangular-shaped (50 mm × 11.5 mm) samples. The three-point bending method was used to step the temperature from room temperature to 120 °C at a rate of 5 °C/min at varied frequencies (1 Hz, 5 Hz, and 10 Hz).

### 2.4. Surface Potential and Charge Trap Characteristics

**Figure 1:**
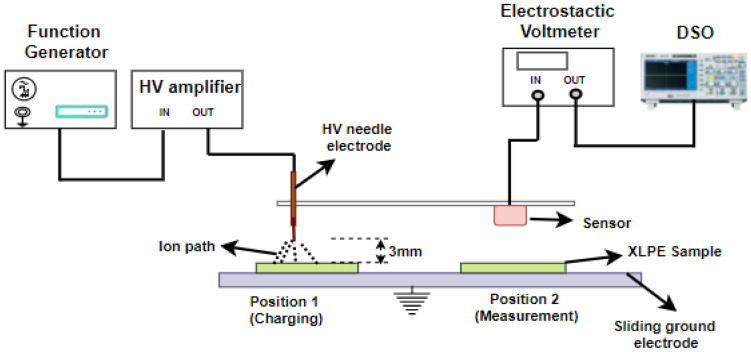
Schematic representation of surface potential decay system.

Figure 1 depicts the surface potential decay system schematically. The charging process uses a needle electrode (0.3 mm tip radius) and a bottom-plane ground electrode (sliding Al sheet). The needle tip is positioned at 3 mm from the sample surface. The needle electrode injects the corona with a 10 kV DC voltage. The charging time is maintained for 3 min. An electrostatic voltmeter (Trek model 341B) continually monitors the surface potential decay after charge injection. Data are then recorded in a real-time digital storage oscilloscope (DSO).

### 2.5. Laser-Induced Breakdown Spectroscopy Experimental Setup

**Figure 2:**
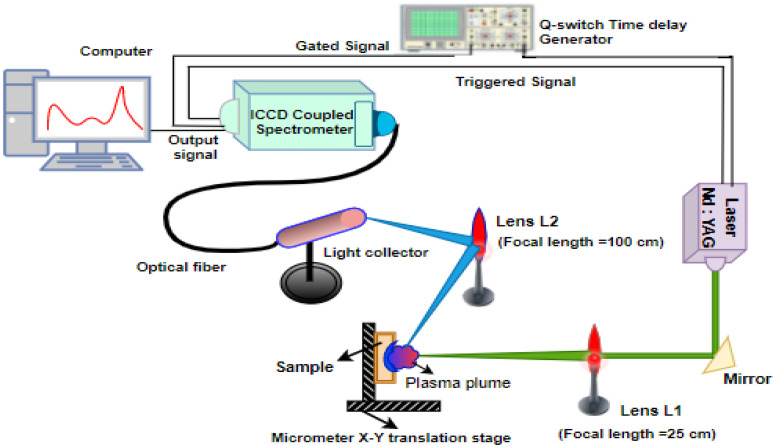
Laser-induced breakdown spectroscopy setup.

As shown in Figure 2, the LIBS setup was employed to assess the elemental and chemical composition of aged and unaged samples. The LASER beam from the pulsed laser source (Nd^3+^: YAG laser (LAB-150-10-S2K, Quanta-Ray LAB series, Spectra-Physics)) is allowed to focus on the sample kept on a controlled motorized stage using a lens of 25 cm focal length to get a plasma plume. The fundamental mode of this laser is at 1064 nm with a pulse duration of 10 ns. The plasma emitted radiation is focused through a lens of 100 cm focal length, and collimated into a spectrometer (Ocean Optics USB2000+UV-VIS-ES) through an optical fiber of core diameter of 400 μm, 0.22 NA. The spectral data were then analyzed in the 200–800 nm wide spectral range. All measurements were performed in air atmosphere.

## 3. Results and Discussion

### 3.1. Water Diffusion Studies

**Figure 3:**
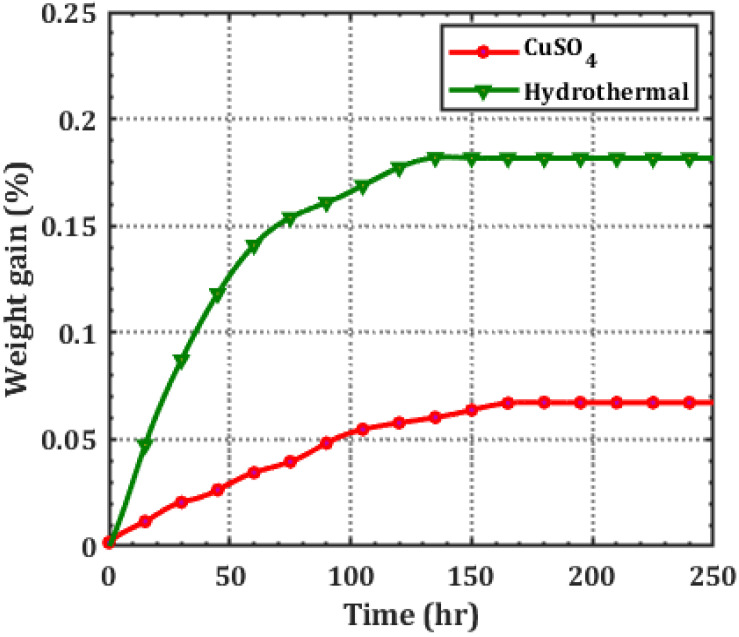
Variation in % weight gain of XLPE specimen due to aging.

The aqueous solution will diffuse into the sample until equilibrium is achieved. Figure 3 depicts XLPE weight growth changes due to hydrothermal and CuSO_4_ aging methods. In order to estimate the diffusion coefficients, weight increase was measured in the test chamber after each aging exposure. The weight gain formula is given as follows:

(2) $$W_{r}\%=\frac{\left(W_{2}-W_{1}\right)}{W_{1}}\times 100$$

where W_2_ and W_1_ are the wet and dry sample weights, respectively. Hydrothermal and CuSO_4_ aged specimens attained equilibrium after 132 and 165 h, respectively. As seen in Figure 3, the initial rate of weight growth was fast, but slowed with time. The proportion of weight growth was directly related to the ion concentration in the material. The diffusion coefficient (D) was calculated by using Equation (3), i.e., [17].

(3) $$D=\frac{\pi L_{0.5}^{2}}{64t_{0.5}}$$

where L_0.5_ is the thickness of the specimen and t_0.5_ is the time of absorption.

The hydrothermal specimen has a higher diffusion coefficient (6.23 × 10^−13^ m^2^ s^−1^) than the CuSO_4_ specimen (3.37 × 10^−13^ m^2^ s^−1^). The ion-conductive clusters operate as polymer diffusion sites. Diffusion is sluggish if the ion-conductive sites are few. The hydrothermally aged specimen’s oxidised (carbonyl) ion clusters are more polar than the unaged specimen’s hydrocarbons alone [18]. As a result, the hydrothermal specimen diffuses more ions than the CuSO_4_ specimen. Thus, the ion diffusion through an oxidized hydrothermal specimen is faster than that of the specimen that has not been oxidized with CuSO_4_.

### 3.2. Contact Angle Investigation

**Figure 4:**
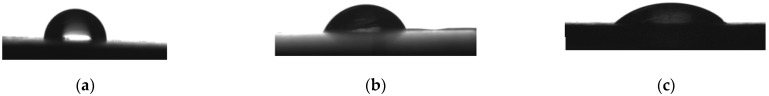
Droplet shape for (**a**) unaged, (**b**) CuSO_4_, and (**c**) hydrothermally aged surfaces.

Water that is repelled by a substance is said to be hydrophobic. Minimal hydrophobicity consequences the increased leakage current, flashover risk, and material degradation [16]. The wettability of solid surfaces is governed by two elements: chemical and geometrical considerations [16]. Figure 4a–c depict typical water droplet position on unaged and aged XLPE specimen surfaces. Figure 4a shows an unaged XLPE specimen with a nearly spherical form and an average contact angle of 88° ± 3.73°. The droplet’s geometric form was flattened for aged specimens, increasing hydrophobicity.

The contact angle for CuSO_4_ aging is 78° ± 6.22°, resulting in an 11% decrease due to the acidic influence of dilute H_2_SO_4_ on hydration. On the other hand, the contact angle of hydrothermally aged specimens drops considerably to 56° ± 6.22° indicating a hydrophilic nature of the specimen. Because of the cumulative impacts of water and temperature influences, hydrothermal aging affects polymer chain re-orientation and increases oxidized (carbonyl) ion clusters, resulting in hydrophobicity loss [18]. LIBS spectroscopic tests, which are described in the next section, are used to determine the concentration of element existence due to diffusion.

### 3.3. Tensile Analysis

**Figure 5:**
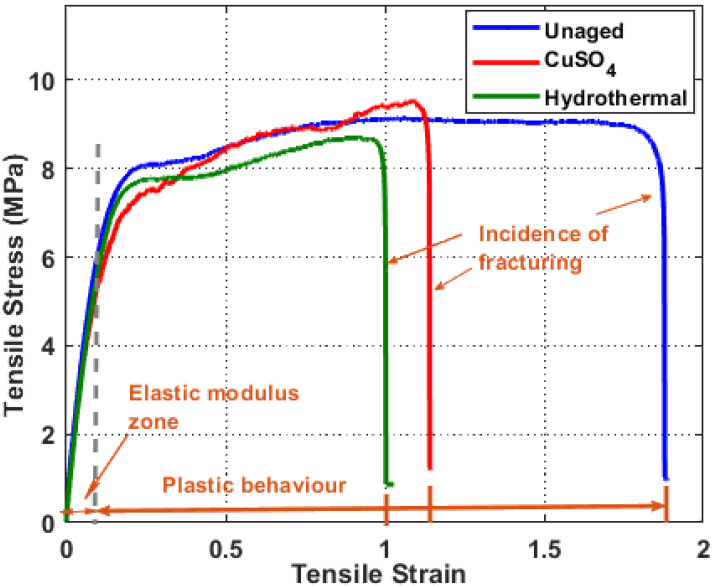
Tensile stress-strain curves for unaged and aged XLPE.

In harsh environments, the mechanical characteristics of insulating polymers degrade before their dielectric properties. As a consequence, in the cable industry, fracture property, tensile strength, and 50% elongation-at-break (EAB) become key metrics for cable evaluation [19]. During wet-aging, moisture penetrates into the cable insulation. As seen in Figure 5, all specimens examined suffered plastic deformation. In the plastic deformation region, the unaged sample exhibited flat stress-strain variation, whereas the aged XLPE showed a linear stress variation against the strain. According to the findings of the tests, tensile strength and EAB seem to be higher for CuSO_4_ aged material than for hydrothermally aged material.

The tensile characteristics retrieved from the stress-strain curves of different XLPE specimens are shown in Table 1. When the material crosslinks with CuSO_4_, a reinforcing effect occurs, and a significant increase in the force needed to cause a rupture compared to the unaged material can be observed; hence, the ultimate tensile strength is higher (9.55 MPa). The CuSO_4_ specimen (9.55 MPa) has the highest ultimate tensile strength, followed by unaged (9.14 MPa) and hydrothermally aged (8.73 MPa). According to the findings, a concurrent decrease in Young’s modulus (51.25 MPa) was also noted as the crosslink density was moderately increased with CuSO_4_ aging compared to the unaged specimen. Unaged samples have a higher Young’s modulus than aged specimens. The increased Young’s modulus for hydrothermally aged specimen may be due to higher crystallinity than the CuSO_4_ aged XLPE.

Highly crystalline materials experience less plastic deformation. When the sample undergoes hydrothermal aging, the materials are more crosslinked, and the polymer chains become restrained, resulting in a decrease in the EAB. The EAB was moderately affected in CuSO_4_ at very low crosslink densities since the few crosslinks present have little effect on the ability of the chains to align in the stress direction. The fracture strength parameter shows no evident difference between the unaged and aged specimens.

Hydrothermal aged samples had a higher creep rate than CuSO_4_ samples. These results influence the tensile properties of the samples, which modifies the internal characteristic of the XLPE material. XLPE insulation’s amorphous cross-linking by-products would disrupt the alignment of macromolecular chains along the stretch direction, reducing tensile strength. However, improving the crystalline structure would enhance XLPE insulation’s mechanical performance [20]. The amount of C–C bonds on the XLPE molecular chains in the amorphous regions determines the elongation at the break of XLPE [18].

### 3.4. Influence of Aging on Trap Parameters of XLPE

**Figure 6:**
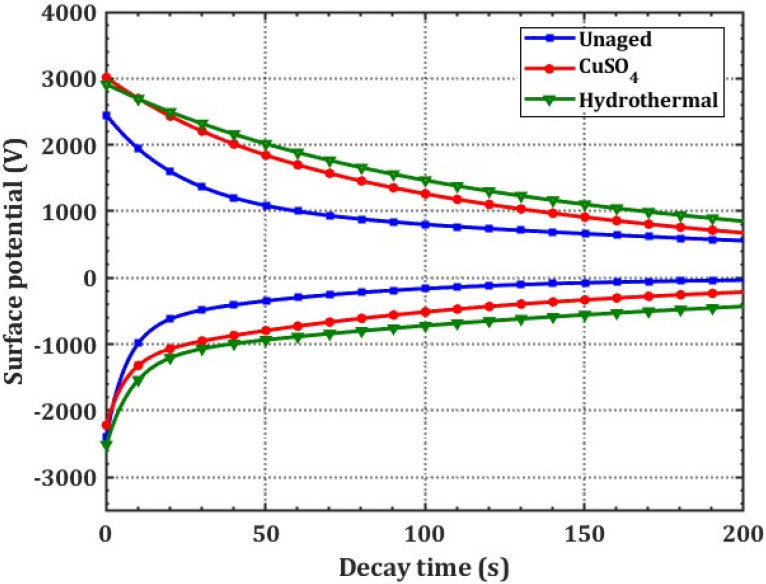
Variation in surface potential decay curves for both polarities.

Corona charging produces electrons and positive ions on the surface. Charge carriers enter the material and become trapped in the localized surface states. Localized states in the material strongly affect charge transfer properties [21]. As detrapping occurs in the absence of applied electric force from the needle electrode, the surface potential of the sample decreases. As shown in Figure 6, the initial potential is more significant under +DC than −DC corona injection. The cause may be ascribed to XLPE’s affinity towards the positive charges. The surface potential decay characteristics are then fitted using an exponentially decaying function given by Equation (4),

(4) $$V\left(t\right)=V_{0}*e^{-\frac{t}{\tau }}$$

where, V_0_ and *τ* are initial potential and decay time, respectively. The decay rate (λ = 1/*τ*) of XLPE specimens decreased with the aging condition. Table 2 shows the decay time of different specimens under ±DC charging conditions. Furthermore, the faster charge decay rate is noticed for the unaged specimen over its aged counterparts. In addition, the decay rate of charges for the hydrothermally aged specimen is slightly slower than CuSO_4_ indicating that electron and hole traps inside this specimen are associated with charge detrap from deep traps.

Figure 6 shows that the energy distribution of charge carrier traps in the material is incongruent. It is therefore necessary to analyze the energy of charge carriers trapped in a specimen. Using surface potential decay (SPD) measurements, trap characteristics, including energy level of carrier trap (E_t_), and trap density N(E_t_) were shown to be strongly connected to decay behavior through the following Equations (5) and (6) [22].

(5) $$E_{t}=E_{c}-E_{d}=k_{B}T\;\ln\left(vt\right)$$

(6) $$N\left(E_{t}\right)=\frac{4\varepsilon_{0}\varepsilon_{r}}{{\mathrm{qL}}^{2}k_{B}T}\left|t\frac{\mathrm{dV}}{\mathrm{dt}}\right|$$

where N(E_t_) is the trap density occupied by carriers at trap level E_t_ in m^−3^ eV^−1^, ε_0_ is the permittivity of vacuum in F/m, ε_r_ is the relative permittivity of the material; q is the unit electron charge (1.6 × 10^−19^ C); k_B_ is the Boltzmann’s constant (1.38 × 10^−23^ J/K); T is the absolute temperature (298 K); L is the thickness of the sample (1.2 × 10^−3^ m), t is the decay time in s, V is the surface potential of the sample in V; v is the escape frequency of the trapped charge carriers, 4.17 × 10^13^ s^−1^.

**Figure 7:**
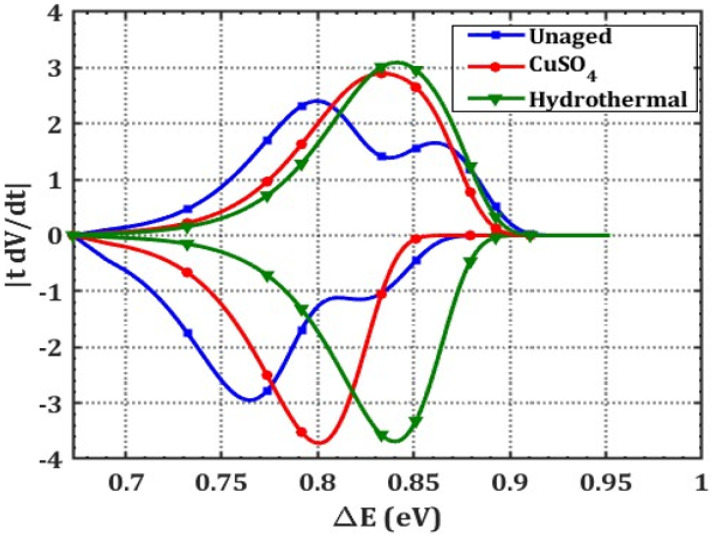
Variation in trap density as a function of trap depth for a ±DC input voltage.

Figure 7 shows electron-type and hole-type traps in the specimens with negative and positive DC inputs. The trap distribution energy ranges from 0.67 to 0.95 eV. Unaged XLPE has two trap centres (ΔE): shallow (0.76 eV) and deep (0.82 eV) traps. Impurities and crosslinking by-products cause shallow traps and space charge buildup [23]. According to unaged XLPE data, electron (0.76 eV) and hole (0.80 eV) trapped energies are bigger than hole-type trapped charges [24]. Table 2 shows the trap properties of the various XLPE samples under study.

In CuSO_4_ aging, hole-type traps dominate the surface states of the sample. For example, under negative polarity, trap depth increases by 5.26% for CuSO_4_ and 10.52% for hydrothermal aging in comparison with the unaged specimens. Ouyang et al. demonstrated that the rupture of XLPE molecular chains during ageing causes alteration in the crystallinity and increased trap densities [25]. Thus, higher trap energy levels are noticed for aged specimens over the unaged sample. Further, the right shift (increased) in trap depth correlates with the rise in surface potential decay time.

### 3.5. Influence of Aging on Viscoelastic Properties

**Figure 8:**
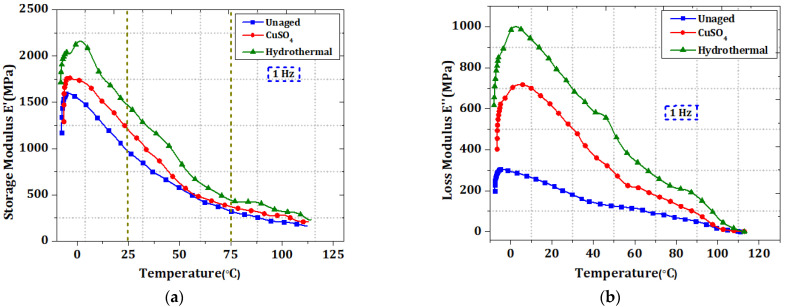

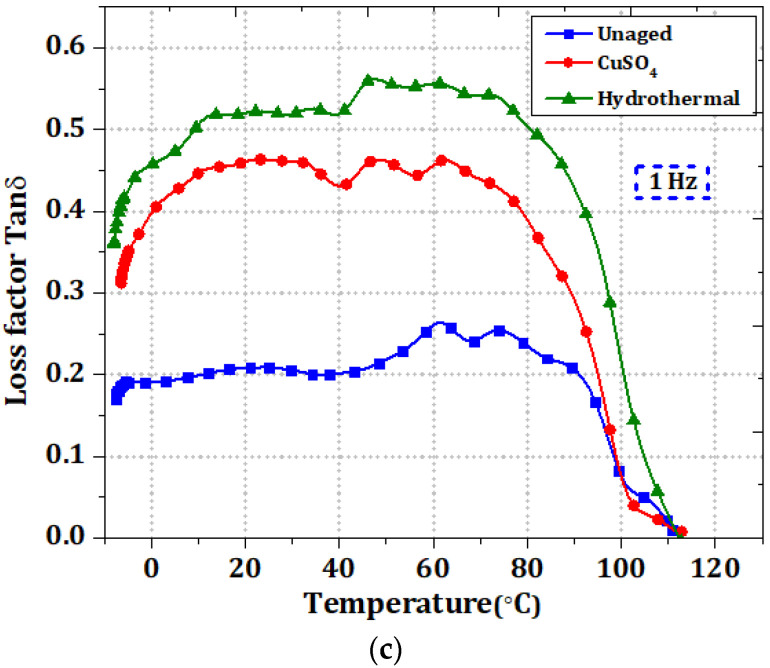
Dynamic relaxation temperature spectra in (**a**) storage modulus E’, (**b**) loss modulus E”, and (**c**) mechanical loss factor for unaged, CuSO_4_, and hydrothermally aged XLPE samples.

Figure 8a–c shows the DMA thermograms for several XLPE materials with storage modulus (E’), loss modulus (E”), and tan δ values. The E’ values decrease with increasing temperature regardless of the specimen type, which is probably connected to crankshaft chain motion [26]. The material’s stiffness diminishes as the temperature increases due to the kinetic motion of molecular chains [27]. The E’ curve varies below 50 °C. In this range, hydrothermally aged specimens have greater E’ values than CuSO_4_ and unaged specimens. A connection exists between crosslinking and crystallinity [26]; aging causes change in cross-linking or crystallinity degree. The aged specimen’s oxidized (carbonyl) ion clusters are more polar than the unaged specimen’s hydrocarbons alone. Because oxidised ion diffusion reduces crosslinking, the storage modulus of hydrothermally aged specimens increases. The storage modulus of CuSO_4_ and unaged specimens has decreased due to reduced crystallinity [26]. In Table 3, E’ for hydrothermal is greater than CuSO_4_, indicating a change in micro-crystalline structure size and alignment.

The transition phase between polyethylene lamellae creates the loss modulus peak for water-aged and CuSO_4_ aged specimens. The most prominent temperature peak in Figure 8c curves describes the twisting and slip of polyethylene folded lamellae crystal molecular chains. The unaged XLPE tan δ curve reveals transitions at 73.9 °C (α relaxation) and 60.9 °C (α’ relaxation). The crystalline segments have a high peak loss factor at 60.9 °C (relaxation). Unaged now displays more significant variations in crystalline segments. CuSO_4_ specimens (Figure 8c) show two transitions at 48 °C (α) and 63 °C (α’), with the relaxation occurring at 48 °C. The ionic solutions caused higher instability in the chain segments of CuSO_4_ aged samples. Significant hydrothermal relaxation occurs at 47 °C. Hydrothermal aging of specimens quickly alters the crystalline segments.

Tiny and defective crystals melt readily even at lower temperatures [27]. Chemicals permeate into the molecular chain during hydrothermal age, removing backbone chain homogeneity and reducing micro-crystalline dimensional changes. The loss modulus peak E” is greater in hydrothermal specimens. The internal friction (tan δ) is measured at 25 °C and 75 °C temperature zones. Unaged and hydrothermal measurements are increased in the RT zone compared to the HT zone in CuSO_4_. A decrease in crystallinity occurs during laboratory ageing of hydrothermal and CuSO_4_, resulting in an increase in crosslinking degree.

**Figure 9:**
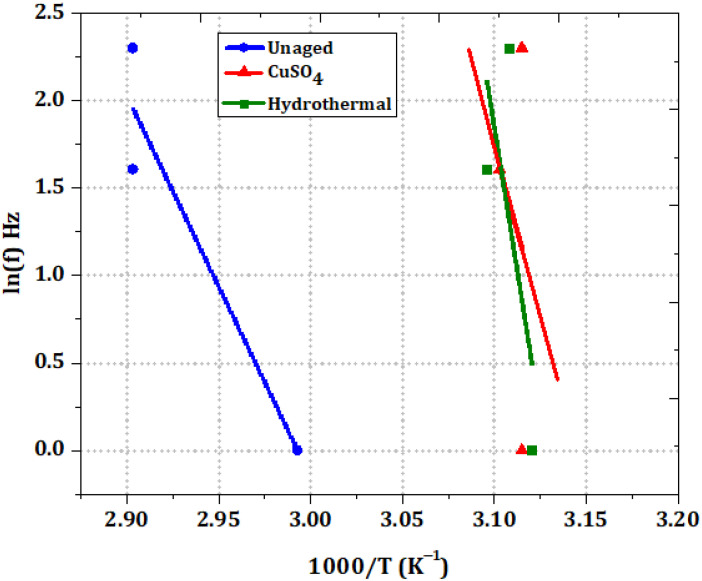
Activation energy plot for unaged and aged XLPE samples.

The transition temperatures (T) presented in Table 3 at different frequencies, and activation energies of (E_a_) of fresh and aged XLPE samples are related by the Arrhenius Equation (7):

(7) $$f=f_{0}*\exp\begin{array}{c} \left[\frac{-E_{a}}{RT}\right] \end{array}$$

where f is the applied vibrational frequency, R is gas constant, and f_0_ is proportionality constant. To determine activation energy, all samples were fitted with an Arrhenius plot between ln(f) and 1000/T, and the plots are shown in Figure 9. The usual value of bond energy for C-H and C-C polymer is 4.0 eV [28]. CuSO_4_ aging has an activation energy of 3.85 eV, which is slightly less than 4.0 eV. Minor charge carriers are found in deep traps from the surface potential studies. Understandably, increasing the activation energy of 5.68 eV would increase the crystallinity.

### 3.6. LIBS Qualitative Analysis

**Figure 10:**
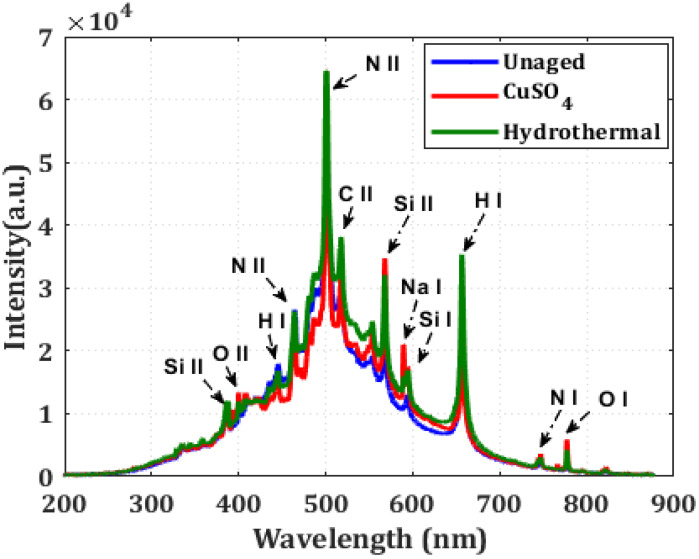
Emission spectra of XLPE sample in the wavelength 200–800 nm.

In this study, the LIBS was used to analyze the composition of aged XLPE samples and classify them based on the intensity of inorganic and organic components in the LIBS spectra. Figure 10 shows the spectral peaks for the elements Si, Na, H, N, and O for the specimens under study at 594.85 nm, 589.50 nm, 656.27 nm, 746.9 nm, and 777.41 nm, respectively. This NIST database was used to assign spectral lines to all XLPE obtained using the LIBS setup. The identification of constituents is dependent on spectral wavelength and intensity [29]. Based on the spectra, the temperature of the plasma (T_e_) formed by laser ablation can be calculated as per the Boltzmann Saha Equation (8) [14],

(8) $$T_{e}=\frac{\left(E_{2}-E_{1}\right)}{k_{B}}{\left[\ln\left(\frac{I_{1}\lambda_{1}g_{2}A_{2}}{I_{2}\lambda_{2}g_{1}A_{1}}\right)\right]}^{-1}$$

where E_1_ and E_2_ are the excited energy levels, g_1_ and g_2_ are the statistical weights of excited energy levels 1 and 2, respectively, A_1_ and A_2_ are transition probabilities of states, I_1_ and I_2_ are the intensities of particular atomic species at λ_1_ and λ_2_ wavelengths, respectively under the condition of local thermodynamic equilibrium [30]. In an insulating material, laser ablation is analogous to localized thermal stress. Unaged, CuSO_4_ and hydrothermally aged XLPE plasma temperatures are 19,300 K, 33,700 K, and 33,300 K, respectively. Principal Component Analysis (PCA) and ANN algorithms were further used to classify the XLPE samples based on the spectral intensity data.

#### 3.6.1. Principal Component Analysis of LIBS Data

In the present study, PCA is used—a feature extraction approach used to categorize LIBS spectral information of different XLPE samples at the first step. It does so by finding larger eigenvectors computed from the data matrix with spectral wavelength as observations (rows) and intensity as a variable (columns). By reducing the dimensions of the original data, PCA can help identify the components with the highest variance. Modified components are sorted by reduced variance. Thus, the first few components describe the entire dataset [31,32]. To identify CuSO_4_ aging from hydrothermal, a dataset of 70 spectra for each sample, constituting a total of 210 spectra, is created, containing 2048 features, and used PCA to separate them.

**Figure 11:**
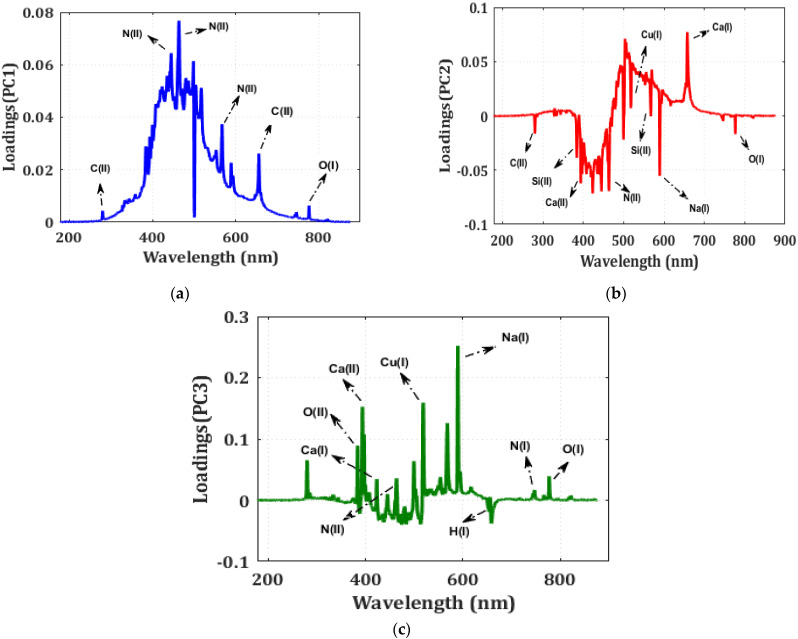
PCA loading plot of unaged and aged XLPE specimens (**a**) PC1, (**b**) PC2, and (**c**) PC3.

**Figure 12:**
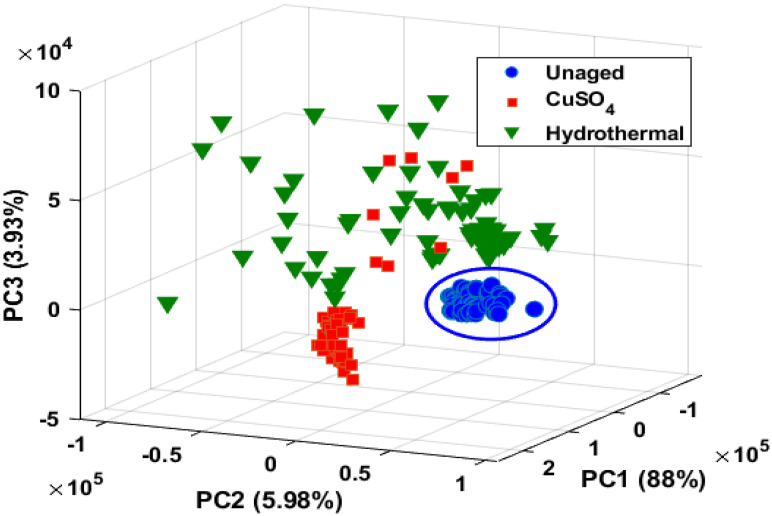
PCA score plot of test specimens classified with respect to the aging studies.

The Principal Component (PC) coefficients, or loadings, indicate the spectral data variances. Figure 11a–c show PCA classification of XLPE utilizing 70 spectral data per specimen. PCA data categorization uses a transformation or score plot. In Figure 12, each spectrum of the specimen is represented by a marker. PC1 (88%), PC2 (5.78%), and PC3 (3.93%) explain the total variance (97.91%) across the data sets (3 × 2048 matrix). The PCA derived from the LIBS spectra of all observed peaks revealed the presence of clusters. The LIBS spectra of unaged XLPE and aged specimens were distinguished; however, the LIBS spectra of CuSO_4_ and hydrothermal specimens had some overlaps. Statistical approaches like PCA are incompatible with LIBS spectra analysis to categorize aged materials. Thus, ANNs were considered for data categorization [31].

#### 3.6.2. Artificial Neural Network Study of LIBS Data

**Figure 13:**
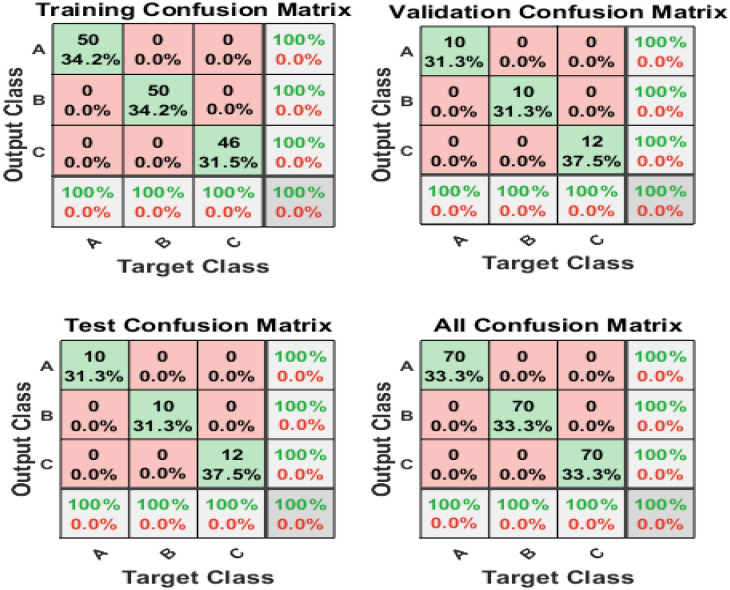
Confusion matrices of XLPE specimens classified with the test spectra of (**A**) unaged, (**B**) CuSO_4_, and (**C**) hydrothermally aged specimens.

ANN was used on LIBS data to characterize aged XLPE based on aging type. The neural network has two layers, one input, one output, and a hidden layer. The input matrix for classifying XLPE specimens depending on aging type consists of 2048 dimensions, whereas 70 samples from each of the three categories constitute a target matrix with a size of 70 × 3. The data matrix is divided into three parts: training, validation, and testing. The present network is trained to utilize a scaled conjugate gradient backpropagation algorithm [31]. Figure 13 shows a visual depiction of the classification model’s performance. Green cells in the confusion matrix represent correctly classified samples, whereas red cells represent misclassified samples [31].

Table 4 shows the superlative cross-entropy performance and epoch for sample categorization based on the aging scenario. The receiver operating characteristic (ROC) is another helpful indicator for assessing sample categorization using a neural network [31]. With 100% accuracy, the ROC of water-aged and CuSO_4_ samples is concentrated on the left-upper half [31]. Thus, it is noticed that the ANN correctly categorizes unaged and aged XLPE in all aging situations with great accuracy.

## 4. Conclusions

This paper investigates the electromechanical and physiochemical characteristics of XLPE material under various aging situations. The following are the key findings from the current study:

- The diffusion coefficient was shown to be more accelerated under hydrothermal conditions than in salt concentrated conditions. The contact angle measurements indicated the increased surface wettability with aging. The diffusion coefficient and contact angle measurements of aged specimens showed an inverse relation.
- Despite a decrease in tensile strength and EAB for aged specimens compared to unaged samples, the CuSO_4_ aged material exhibited improved properties over the hydrothermally aged materials.
- On charge deposition over the surface of the insulating material, irrespective of polarity, the surface potential variation exhibited exponential decay behaviour. The range of trap energy distribution for an XLPE material lies in the region of 0.67 eV to 0.95 eV. Also, it was observed that the trapped energy levels are different for positive and negative deposited charges. Surface potential measurements for CuSO_4_ and hydrothermally aged specimens showed decreased decay rates when compared to unaged samples. The deep trap energy sites of both electron and hole-type grew dramatically in the aging scenario.
- The DMA results revealed that aged specimens had a lower glass transition temperature and higher loss tangent than unaged counterparts.
- Hydrothermal specimens had the highest level of oxidation and hydrophilicity levels, which contributed to the low life of the XLPE insulation. The LIBS method was used for the qualitative analysis of the materials used in the manufacture of cable insulation. The level of aging was qualitatively classified with a higher accuracy using ANN compared to the principal component analysis.

Therefore, there is a strong prerequisite to address these problems for the benefit of cable utilities in addition to the cable manufacturers.

## Figures and Tables

**Table 1:** Variation of tensile properties of XLPE.

Sample	P (N)	Q (MPa)	R (MPa)	S	T	U (MPa)	V (mm/sec)
Unaged	253.37	69.53	9.14	112.4	187.74	8.85	0.209
CuSO_4_	233.24	51.25	9.55	108.6	114.06	8.77	0.048
Hydrothermal	221.78	54.13	8.73	92.11	100.2	8.49	0.257

P—Load at maximum tensile stress. Q—Young’s modulus, R—Ultimate tensile strength (UTS). S—% Strain corresponds to UTS; T—% Elongation at break; U—Fracture strength; V—% Creep rate.

**Table 2:** Mean lifetime and trap parameters of XLPE under DC voltage.

Sample	Positive Polarity		Negative Polarity	
	Decay Time (s)	Trap Depth, (eV)	Decay Time (s)	Trap Depth, (eV)
Unaged	76.00	0.80	12.60	0.76
CuSO_4_	119.60	0.83	47.20	0.80
Hydrothermal	155.80	0.84	52.80	0.84

**Table 3:** Mechanical loss factor and storage modulus of various XLPE samples.

Sample/ Parameters	Storage Modulus (E’) (MPa)		Loss Modulus (E’’) (MPa)		Tanδ		Transition Temperature, T_g_ (K) at Different Frequencies			Activation Energy E_a_ (eV)
	RT (25 °C)	HT (75 °C)	RT (25 °C)	HT (75 °C)	RT (25 °C)	HT (75 °C)	1 Hz	5 Hz	10 Hz	
Unaged	968	321	202.09	81.49	0.21	0.25	334.14	344.46	344.46	1.88 eV
CuSO_4_	1218	374	550.33	159.42	0.46	0.43	321.03	322.27	321.03	3.29 eV
Hydrothermal	1469	437	766.88	240.59	0.52	0.53	320.49	323.01	321.74	5.68 eV

**Table 4:** Cross-entropy performance of classified samples.

Process	No. of Samples	Cross-Entropy Error (CE)	Minimum Error during Validation	Epoch at Minimum Error
Training	146	1.389		
Validation	32	4.127	7.446 × 10^−3^	31
Testing	32	4.082		

## Data Availability

Not applicable.
